# Infection Masquerading as Recurrence of Pancreatic Ductal Adenocarcinoma: A Cautionary Tale

**DOI:** 10.7759/cureus.17010

**Published:** 2021-08-08

**Authors:** Sowbharnika Arivazhagan, Deepti Kantamani, Natalee E Tanner, Madappa N Kundranda, M. Patrick Stagg

**Affiliations:** 1 Internal Medicine, Baton Rouge General Medical Center, Baton Rouge, USA; 2 Pharmacy, Banner MD Anderson Cancer Center, Gilbert, USA; 3 Hematology and Oncology, Banner MD Anderson Cancer Center, Gilbert, USA; 4 Internal Medicine Residency Program, Baton Rouge General Medical Center, Baton Rouge, USA

**Keywords:** ca 19-9, pancreatic adenocarcinoma, infectious causes of ca 19-9, liver abscess, positive predictive value of ca 19-9

## Abstract

We present a case of a 59-year-old male undergoing adjuvant chemotherapy for his pancreatic adenocarcinoma post-surgical resection. He had an acute rise in carbohydrate antigen (CA) 19-9 level, which raised suspicion of metastatic disease. Instead, the patient was diagnosed to have a liver abscess, the treatment of which brought the CA 19-9 level back to normal. Unfortunately, although CA 19-9 is Food and Drug Administration (FDA)-approved tumor marker for pancreatic cancer, it is also elevated in several benign conditions, causing fear of cancer and unnecessary diagnostic workup. Hence, caution is necessary for interpreting the significance of its elevation.

## Introduction

Pancreatic cancer is the fourth leading cause of cancer deaths in the United States. More than 2000 studies have analyzed various biomarkers for improving the detection and monitoring of pancreatic cancer [1]. Cancer antigen (CA) 19-9 is the most extensively studied and only validated tumor marker for pancreatic cancer and is approved by the Food and Drug Administration (FDA) [2-3]. In pancreatic cancer, CA 19-9 overuse as a diagnostic tool in symptomatic patients to assess tumor stage and resectability, as a biomarker of prognosis following resection, to assess chemotherapy response, and as a predictor of postoperative response is common [4]. National Comprehensive Cancer Network (NCCN) guidelines recommend CA 19-9 testing at the time of diagnosis and for post-treatment surveillance. NCCN also emphasizes that elevated CA 19-9 post-treatment does not always indicate cancer recurrence and that elevated levels may be due to benign conditions. The American Society of Clinical Oncology does not recommend its use for the above as there is no validation of standardized cut-off values, except for CA 19-9 levels >37 used in diagnosis [4-5].

The CA 19-9 has poor sensitivity, false negatives in Lewis blood group negative phenotype (5%-10%), high false positives in the presence of obstructive jaundice, and non-specific expression in several benign diseases limits its role as a screening tool in cancer detection [2]. In symptomatic patients, it has a sensitivity of 79%-81% and specificity of 82%-90% for diagnosing pancreatic cancer and a very low positive predictive value (PPV) of 0.5%-0.9% [2]. The CA 19-9 has no role in screening asymptomatic patients. Kim et al. found that only four Patients, among 1,063 patients with elevated CA 19-9 had pancreatic cancer in a screening study of asymptomatic patients [2]. Satake et al. found only four pancreatic cancers among 18 asymptomatic patients with elevated CA 19-9 levels [2]. Chang et al. have identified only two patients with pancreatic cancer among 385 asymptomatic patients with elevated CA 19-9 [2]. PPV is 0.5% in asymptomatic patients, so screening with CA 19-9 has no clinical utility [2]. CA 19-9 has a higher predictive value for diagnosing pancreatic cancer in patients who present with a pancreatic mass [2]. CA 19-9 levels are predominantly used in practice to assess the prognosis after neoadjuvant therapy with or without resection and to predict postoperative response [2]. In resectable disease, a decrease in CA 19-9 postoperatively is indicative of more prolonged survival. A decrease in CA 19-9 after treatment may signify better response and prolonged survival in advanced disease. In the case presented here, the marker was acutely elevated eight months after the surgery during the fifth cycle of chemotherapy, raising suspicion for recurrence of malignancy. Instead, it was because of an infection, and it dropped down appropriately with the treatment.

## Case presentation

A 59-year-old male with resectable pancreatic ductal adenocarcinoma, and a baseline CA 19-9 of 241 IU/mL, underwent a pancreaticoduodenectomy. The pathology demonstrated a 2.8 cm moderately differentiated pancreatic ductal adenocarcinoma extending to the peripancreatic soft tissue. Margins were negative with no lymphovascular or perineural invasion. One lymph node of the 23 removed was positive; surgically, the staging was pT2N1. The patient was started on adjuvant gemcitabine and capecitabine. A CT of the chest, abdomen, and pelvis before adjuvant therapy did not show any evidence of metastatic disease, and CA 19-9 measured 22.2 IU/mL. After three months of treatment, repeated staging CT did not reveal any metastatic disease, despite the interval increase in CA 19-9, increasing to 42.8 IU/mL. Thus, the patient continued with his last three months of adjuvant treatment.

 At the start of the last cycle (fifth cycle) of adjuvant treatment, his CA 19-9 had increased to 158.7 IU/mL, which raised the suspicion of metastasis. However, during his last cycle, the patient developed a fever and presented to the emergency room (ER) after completing five cycles of adjuvant chemotherapy. 

**Figure 1:**
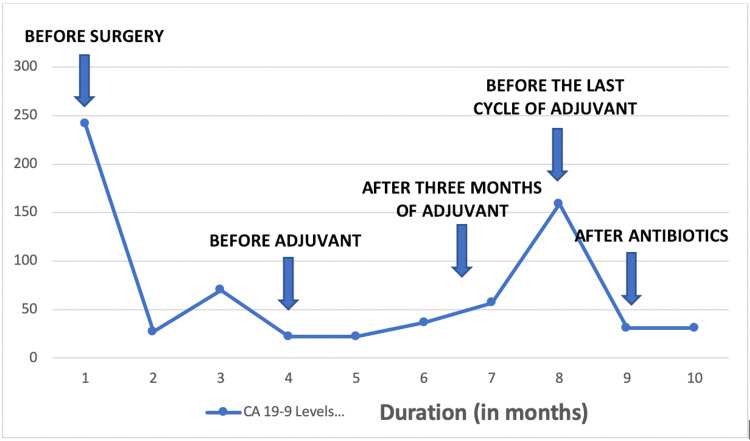
CA 19-9 levels during various stages of treatment. X-Axis - duration in months; Y-Axis - CA 19-9 levels in IU/mL CA 19-9, carbohydrate antigen 19-9

A CT of the chest, abdomen, and pelvis demonstrated a new 4.4 cm x 3.6 cm mass involving segment 4 of his liver along with other lesions involving segment 4B. Despite the rise in the CA 19-9, this appeared to be more consistent with a liver abscess than with new metastatic disease. An ultrasound-guided biopsy for diagnosis provided tissue for pathology that showed necroinflammatory debris with degenerative liver tissue without any evidence of metastatic disease. Culture from the biopsy was positive for Escherichia coli. As a result, the patient's last cycle of chemotherapy was not given. He was started on IV piperacillin and tazobactam (extended release) with a 3.375 g in 100 mL infused 25 mL/h. After completing the antibiotics, the CA 19-9 decreased to 31.0 IU/mL, and a repeat CT of the abdomen and pelvis showed near-complete resolution of his liver abscess. The time course of the patient's CA 19-9 level is shown in Figure 1. 

## Discussion

Carbohydrate antigen 19-9 (CA 19-9) is a sialylated Lewis blood-group antigen first described by Koprowoski et al. in 1979 using a mouse monoclonal antibody (1116NS 19-9) in a colorectal cancer cell line [2]. Disialyl Lewis-a is normally expressed on the epithelial cells of the digestive organs, and it helps in immune surveillance. Epigenetic silencing of sialyl transferase during the early stages of carcinogenesis leads to abnormal synthesis and accumulation of sialyl Lewis-a, commonly referred to as CA 19-9 [2, 6]. In locally advanced cancers, it is postulated that tumor-related hypoxia triggers transcription of several glycogenes resulting in excessive CA 19-9 [7]. 

The CA 19-9 is a serum tumor marker for gastrointestinal malignancies, especially of the pancreas and biliary tract. It can also rise in several benign diseases of the hepatobiliary tree, lung, kidney, endocrine, and connective tissue as shown in Table 1. The mechanism of elevated CA 19-9 levels in benign conditions is associated with inflammation. 

**Table 1:** Conditions associated with elevated levels of CA 19-9. CA 19-9, carbohydrate antigen 19-9; GI, gastrointestinal

Hepato-biliary diseases [10-11]	GI malignancies [1, 4, 6, 12]	Pancreatic conditions [1, 13-14]	Miscellaneous [15-16]
Liver abscess	Gastric cancer	Pancreatic abscess	Bronchitis
Liver cyst	Colorectal cancer	Pseudocyst of pancreas	Cystic fibrosis
Choledocholithiasis	Esophageal cancer	Acute and chronic pancreatitis	Hashimoto's thyroiditis
Cholelithiasis			Ovarian cyst
Hepatitis			Renal cyst
Polycystic liver disease			Congestive heart failure
Cholangiocarcinoma			Diverticulitis
Cholangitis			Lung cancer
Cirrhosis of liver			Pleural effusion
Hepatocellular carcinoma			Rheumatoid arthritis

The CA 19-9 is acutely elevated secondary to a liver abscess in the case presented here, and it normalized with treatment. Inflammation in the biliary tree can induce increased CA 19-9. Similar to this case, there have been additional reports of elevated CA 19-9 with liver abscess [8-9]. CA 19-9 can also be used as a marker to assess response after treatment with antimicrobials in patients with liver abscesses. Case reports from the past describe that elevations in CA 19-9 >40 U/mL are uncommon in benign conditions [5]. However, few benign conditions involving the liver, pancreas, and biliary tract have shown extremely high CA 19-9 levels. 

## Conclusions

The CA 19-9 has relatively high sensitivity and specificity for symptomatic pancreatic cancer. It plays a vital role as a prognostic and predictive marker of treatment response. Nevertheless, nonspecific elevation in several malignant and benign conditions confounds its interpretation, further confounded by false negatives in Lewis negative genotype. Fluctuations in CA 19-9 levels need a thorough evaluation of the complete clinical picture. Any uncertainty about CA 19-9 levels may require imaging and tissue sampling as demonstrated in this case.
